# Musculoskeletal injuries in basketball players Southern Portugal: Epidemiology and risk factors

**DOI:** 10.14744/nci.2021.21549

**Published:** 2022-02-08

**Authors:** Beatriz Minghelli, Sofa Queiroz, Inês Sousa, Julia Trajano, Sara Graça, Vera Silva

**Affiliations:** 1 School of Health Jean Piaget Algarve, Piaget Institute, Silves, Portugal; 2 Research in Education and Community Intervention

**Keywords:** Basketball, epidemiology, injury, sport

## Abstract

**Objective:**

Basketball consists of a contact sport that involved actions such as running, jumps, and abrupt changes in direction several times and these repetitive movements can lead to injuries. The objective of this study was to verify the epidemiology of musculoskeletal injuries and risk factors in basketball players.

**Methods:**

The sample comprises 361 basketball athletes Southern Portugal, being 238 (65.9%) males, aged between 10 and 53-years-old. The instrument used for data collection was a questionnaire with questions about the population characterization and related to the basketball practice and injuries.

**Results:**

Two hundred and thirty-nine (66.2%) players referred an injury throughout their practice, totaling 494 injuries, and 174 (48.2%) players reported an injury in the previous year, with a total of 244 injuries. There were 2.72 injuries/1,000 h of basketball training. The most common injuries were sprain (43.8%), the most injured body area was the ankle (40.1%) and the principal injury mechanism was the impact with another athlete (19.4%). The basketball players who trained more than 4 times a week showed a 2.21 greater chance to develop injury (95% CI: 1.3–3.5; p=0.003) than those who trained less often.

**Conclusion:**

This study showed a high prevalence of injuries in this analyzed sample, being the ankle and knee the most injured body areas, the sprain the more prevalent type of injury, and the impact with another player the main mechanism of injury. The data obtained can be used to create training programs with the objective of preventing injuries on basketball players.

**B**asketball is a popular game practiced all over the world, which is played as a team by people of all the age groups and genders [1–5]. Basketball is a contact sport characterized by movements such as running and deceleration, repetitive jumps, and the respective impact of landing, turns, lateral movements, and abrupt changes in direction. These actions may result in a greater risk of musculoskeletal injuries in this sport [1, 6, 7].

Data of injury rates on basketball reported values between 7 and 10 injuries/1,000 athletic exposures [8]. Andreoli et al. [6] published a systematic review using eleven articles and their data revealed a total of 12,960 injuries.

Zuckerman et al. [9] evaluated 152 basketball players of National Collegiate Athletic Association between 2009 and 2015 seasons and reported a total of 2,308 injuries in men and 1,631 injuries in women’s players, and an injury rates of 7.97 and 6.54/1,000 athlete-exposures in men and women, respectively. Non-time-loss injuries accounted for more than 50%. The lower extremity was the most injured anatomical body area and the most common injury were sprains, strains, and contusions.

Sports injuries lead to a negative impact on athlete’s performance and their sports participation as well as daily activities, reduction in training time, an increase in health expenses, and the risk of new injuries [1, 2, 7].

Although there are several international studies on the epidemiology of basketball injuries, some studies focus only on a specific type of injury, others studies involve only one gender, and others only one type of competitive level. Besides that, there are few studies carried out in Portugal, and the anthropometric differences of the Portuguese basketball players, as well as the disparities in the rules of the game in this country, compared to the reality of the rest of Europe and especially the United States and Canada, are different. These factors may contribute to a different scenario of basketball injuries between countries, justifying the relevance of this study. Thus, this study aimed to verify the epidemiology of musculoskeletal injuries and risk factors in basketball players living in Portugal since there have unknown epidemiological studies to date.

## Materials And Methods

The nature of this study was cross-sectional to obtain data about musculoskeletal injuries in basketball athletes living in the South of Portugal.

The Research in Education and Community Intervention research center approved this research, as well as the Basketball Clubs Direction. All study participants signed a written informed consent form. In case the athlete is under 18 years, the consent form was signed by the legally responsible person.

### Population

The study population included competitive basketball athletes of all sexes with aged equal or over 10 years (children were excluded).

The research inclusion criteria defined athletes who practiced this sport for a period ≥6 months, who had attended at least 2-week training session, who are present at the time the data was collected, who want to voluntarily participate in the study and who signed the informed consent form.

There are eleven basketball clubs in the South of Portugal. Excluding under-8 and under-10 categories, the population consists of 1,101 basketball players (under 12 n=173, under 14 n=351, under 16 n=271, under 18 n=176, seniors n=130). An estimated mean injury, with a prevalence of 50% reported in international studies [1, 10], was used to determine the sample size, assuming an error margin of 5% with 97% confidence interval (CI). From this approach, the minimum sample size was established in 331 players [11].

**Highlight key points**

• Our data showed a high prevalence of musculoskeletal injuries in basketball players.
• The ankle and knee are the most injured body areas, the sprain is the more prevalent type of injury, and the impact with another player the main mechanism of injury.
• The player who trained more times per week has more probabilities of injury.

### Measurement Instrument

The measurement instrument was applied during the training sessions and consisted of a questionnaire divided into two parts: 1) the socio-demographic characterization of the population and the sport characteristic (frequency and duration of training; years of practice) and 2) specific questions about injuries (occurrence/presence of injuries). The presence of injuries was evaluated in four periods: on the day of the assessment, 6 months ago, 12 months ago, and throughout basketball practice.

Since validated questionnaires on basketball injuries are unknown, this questionnaire was elaborated and analyzed by a group of experts from different areas of expertise (PhD Physiotherapy, coach, and a player with years of practice). Subsequently, a pre-test was applied to 10 athletes.

The measure instrument was applied through an interview conducted by the research, only once. The application of the questionnaire through an interview allowed the investigator to clarify any doubts that might arise.

The athlete who answered that he had an injury in the last 12 months should continue filling out the questionnaire answers questions related to the characteristics of the injuries suffered: injuries number, type, anatomical site and mechanism; the occurrence moment; the treatment performed, and if so, the athlete should state which treatment applied; the inactivity time (lost time of training) and current situation of injury. If the athlete had four or more injuries, only 3 injuries were selected by the investigator to specify the characteristics, taking into account the severity and/or the longest recovery time. The categories of the defined variables are shown in the results section.

The definition of injury consisted of any symptom or condition caused by the practice of basketball, either during training or competition. The injury should have less of one of the following consequences: the athlete failed or was removed, at least 1 day, from training or competition; did not have to stop training, but only managed to adapt the training, with changes in the frequency, duration and/or intensity of training, or with adaptations of technical managements; performed some type of treatment with health professionals to treat the injury [12].

### Injury Proportion and Injury Rate

The injury proportion calculation was made by dividing the number of athletes who suffered at least one injury in the last year (12 months) by the sample number. In order to obtain the injury rate value, a division was made between the total number of injuries of all athletes by the total time that these athletes were exposed to this risk of injury, defined in 1000 h. This total time of injury risk was calculated by multiplying the average total hours of training by the frequency training, both over a period of 1 week. Then this value was multiplied by 12 months (52 weeks) [13].

The injuries number average per athlete was calculated by a division of the total injuries number by the total sample number. The division of the total injuries number by the total number of injured athletes determined the average of injuries per injured athlete [13].

### Data Analysis

The software used to perform the static analysis of the data was the Statistical Package for Social Sciences, version 24.0.

In the first approach, descriptive statistics were performed. The Chi-square independence test was applied to relate the different periods of injury presence with age groups. The binary logistic regressions (Enter methods) were applied to test the influence of the variables used in this study on the injury presence. After, a final multivariate model was developed (Forward Likelihood Method), being the CIs calculated. The validity, quality of fitting, and predictive capacity of the binary logistic regressions were evaluated by the Omnibus test and the Nagelkerke correlation coefficient. The level of statistical significance was established for 0.05.

## Results

The sample was constituted by 361 basketball athletes, aged between 10 and 53-years-old (14.66±5.16), being 238 (65.9%) males and 123 (34.1%) females.

Regarding the position of the player, 58 (16.1) were the point guard, 51 (14.1%) shooting guard, 73 (20.2%) small forward, 21 (5.8%) power forward, 22 (6.1%) center and 136 (37.7%) had no definite position.

Seventy-seven (21.3%) athletes performed another type of sport beyond basketball.

Table 1 shows the years of modality practice. The mean training frequency per week was 3.18 (SD: 0.59) and the training duration per week was 4.5 (SD: 1.04) h.

**Table 1.** Years of basketball practice

Years of modality practice	%
Between 1 and 2 years	29.4
Between 3 and 4 years	30.7
Between 5 and 6 years	19.7
Between 7 and 8 years	8
Between 9 and 10 years	5
More than 10 years	7.2

The prevalence of injury divided by age group is shown in Table 2. We can observe a high prevalence of injury in the whole practice of basketball (66.2%), totaling 494 injuries. One hundred-seven (44.8%) athletes reported one injury since they began their basketball practice, 57 (23.8%) referred two injuries, 27 (11.3%) referred three injuries, and 48 (20.1%) four or more injuries.

**Table 2.** Prevalence of injury in basketball players by age group

Period of injury/Age group	Throughout practice		At the moment		6-month period		12-month period	
	Absence (%)	Presence (%)	Absence (%)	Presence (%)	Absence (%)	Presence (%)	Absence (%)	Presence (%)
10–17 years (adolescents)	38.2	61.8	91.1	8.9	65.6	34.4	52.9	47.1
18–29 years (younger adults)	5.1	94.9	84.6	15.4	53.8	46.2	43.6	56.4
30–60 years (older adults)	0	100	62.5	37.5	62.5	37.5	50	50
P-value	≤0.001	0.017	0.350	0.547				
Total	122 (33.8%)	239 (66.2%)	283 (78.4%)	37 (10.2%)	232 (64.3%)	129 (35.7%)	187 (51.8%)	174 (48.2%)

In the 12-month period, 244 injuries were accounted on 174 athletes. One hundred and nineteen (68.4%) athletes reported one injury, 42 (24.1%) referred two injuries, 11 (6.3%) reported three injuries and 2 (1.1%) athletes reported four or more injuries in the period of 12 months. Since two athletes had more than three injuries and only a maximum of three injuries was allowed to answer, all of the following results will report a total of 242 injuries.

The value of injury proportion was 0.48 (CI 95%: 0.43–0.53) and the injury rate was 2.72 injuries/1,000 h of basketball training.

The average number of injuries per player was 0.68.

The average of injuries per injured player was 1.40.

The values obtained on the type and anatomical site of the measured lesions are presented in Table 3.

**Table 3.** Type and location of injury

Type of injury	Location of injury	Number	%
Fracture	Face	1	
	Arm	2	
	Wrist	3	
	Hand/Fingers	7	
	Thigh	3	
	Leg	2	
	Foot/Toes	8	
	All	26	10.7
Muscle injury (strain, contusion)	Face	2	
	Shoulder	2	
	Arm	1	
	Wrist	2	
	Thigh	8	
	Knee	4	
	Leg	12	
	Ankle	3	
	Foot/Toes	4	
	Lumbar spine	1	
	All	39	15.7
Meniscal injury	Knee	8	
	All	8	3.3
Strain	Shoulder	3	
	All	3	1.2
Sprain	Elbow	2	
	Wrist	13	
	Knee	3	
	Ankle	88	
	All	106	43.8
Tendinopathy	Shoulder	4	
	Knee	6	
	Ankle	4	
	All	14	5.8
Ligament injury	Wrist	4	
	Knee	9	
	Ankle	1	
	All	14	5.8
Low back pain	Lumbar spine	9	
	All	9	3.7
Non-specific pain	Thorax	1	
	Shoulder	2	
	Elbow	2	
	Wrist	1	
	Thigh	1	
	Knee	9	
	Leg	3	
	Ankle	1	
	Foot/Toes	3	
	All	23	9.1
Any type of injury	Face	3	1.2
	Thorax	1	0.4
	Lumbar spine	10	4.1
	Shoulder	11	4.5
	Arm	3	1.2
	Elbow	4	1.7
	Wrist	23	9.5
	Hand/Fingers	7	2.9
	Thigh	12	5.0
	Knee	39	16.1
	Leg	17	7.0
	Ankle	97	40.1
	Foot/Toes	15	6.2
Total		242	100

Athletes suffered the most injuries during training (155; 64.1%); 77 (31.8%) in the context of competition; 9 (3.7%) during the warm-up period, and 1 (0.4%) during the warm-down.

Table 4 shows the mechanism of injury in the period of 12-month.

**Table 4.** Mechanism of injury

Injury mechanism	n	%
Athlete impact	**47**	**19.4**
Fall	34	14
Ball impact	23	9.5
Speed running	22	9.1
Landing	18	7.4
Bounce	15	6.2
Sudden stop	15	6.2
Jump	14	5.8
Repeated motion	10	4.2
Receiving a pass	8	3.3
Defense	5	2.1
Launch	3	1.2
Execution of a pass	2	0.8
Other	19	7.9
Do not know	7	2.9

Two hundred and five (84.7%) athletes were treated. Among these, 118 (57.6%) were treated with physiotherapy; 54 (26.3%) rested or medication; 31 (15.1%) were immobilized for a period of time; and 2 (0.9%) were submitted to surgery.

The athletes were asked about the time they had to interrupt their basketball practice because of injuries, and 15 (6.2%) injuries took the athletes to stop their practice until 2 days, 46 (19%) did interrupted their practice between 3 and 7 days, 40 (16.5%) between 8 and 14 days, 44 (18.2%) between 15 and 30 days, and 53 (21.9%) for more than 30 days and 44 (18.2%) injuries did not take the athletes to interrupt their practice.

About the current situation of the injury, 191 (78.9%) injuries were reported by the athletes to be fully recovered, 9 (3.7%) without pain, but in treatment, 17 (7%) with pain and underwent some type of treatment, and 25 (10.3%) with pain but does not perform any treatment.

The relationship between the injury presence in a period of 12 months and the variables analyzed in this study are shown in Table 5. The position that the athlete takes on the court was divided by the athletes who play outside the court (point guard, shooting guard, and small forward) and those who play inside (power forward and center). The athletes who had no definite position were excluded from this analysis considering the player position. The cuts of the numeric variables age, frequency, and duration of training took into account the values of median and mode.

**Table 5.** Relationship between the event the presence of injury and variables about non-modifiable sample factors and basketball practice characteristics

Variables	Odds ratio_crude_ (CI 95%); p	Odds ratio_Adjusted_** (CI 95%); p
Gender (male*) female	1.27 (0.6–2.5); 0.483	–
Age group (until 13-years-old*) ≥14-years-old	**1.57 (1.0–2.4); 0.034**	–
Years of practice (until 4 years*) ≥5 years	**1.64 (1.1–2.5); 0.023**	–
Weekly training (until 3 times*) ≥4 times	2.27 (1.4–3.7); 0.001	2.21 (1.3–3.5); 0.003
Duration of training per week (until 4 h*) ≥5 h	1.39 (0.9–2.1); 0.132	–
Dominant limb (right*) left	1.08 (0.6–2.1); 0.809	–
Practice of another sport (no*) yes	1.21 (0.7–2.0); 0.458	–
Position (outside the court*) inside the court	1.27 (0.6–2.5); 0.483	–

*: Class reference; **: Forward LR model; CI: Confidence interval.

The final model obtained mathematical validity (Omnibus p=0.001 and Nagelkerke R^2^=0.04).

It was found that basketball players who trained four or more times per week showed 2.12 more probability of developing injury (95% CI: 1.3–3.5; p=0.003) compared to athletes who trained until three times a week.

## Discussion

These main data obtained in our retrospective study revealed that 66% of basketball athletes referred to have an injury during their whole practice and 48% suffered at least one injury in the last year. Leppänen et al. [5] evaluated 207 male basketball players, aged 16 years, and observed lower prevalence values compared to the values obtained in our study, revealing that 80 (39%) players had an overuse injury within a 12-month period. Moreira et al. [2] reported a prevalence injury value of 58.3% in the past 8 months (different period of this study) in 410 male master players, aged 35–85 years (mean 52.26). This high prevalence could be explained due to the great physical requirement of basketball practice, demanding an intense effort from the athletes during training and competition.

The number of injuries obtained in this study is also high (494 injuries throughout the practice and 244 injuries in 12-month period). Caparrós et al. [14] analyzed 44 players from a Spanish basketball club (mean age: 27.6) and reported 162 injuries during seven consecutive seasons (2007/2008 to 2013/2014). Vanderlei et al. [1] evaluated 204 basketball players (mean age: 14.33) and 40 players injured, totaling 46 injuries. Owoeye et al. [15] also evaluated 141 adolescent basketball players (mean age: 16.3) who participated in a total of 32 matches and 32 injuries were recorded during the competition. Foss et al. [16] reported a total of 84 injuries in middle-school girls basketball players in the season of 2009–2010. The injuries number in these studies was accounted in different periods, making comparisons between studies difficult.

Most of our sample included adolescents that are still skeletally immature and show an increased risk of injury due to instability between neuromuscular control, flexibility, and muscle strength [15, 16]. However, the prevalence values obtained in adults in the different periods analyzed in our study are much higher than those obtained in adolescents.

Injury rates can be calculated in several ways, which affect the results of the studies and compromise their comparison because it leads to different interpretations. Our study revealed an injury rate of 2.72 injuries/1,000 h of basketball training. Foss et al. [16] revealed an overall rate of injury of 2.24/1,000 athlete exposures for practice. Clifton et al. [17] reported a total injury rate of 1.82/1,000 athlete exposures in high school basketball and of 4.96 in collegiate players, both female. Borowski et al. [18] collected basketball-related injury data during the 2005–2006 and 2006–2007 academic years from 100 nationally representative US high schools and reported an injury rate of 1.40/1,000 athlete exposures during practice. Weiss et al. [19] registered an overuse injury rate per 1,000 h of athlete exposure of 6.4 on 13 athletes of a professional male basketball players.

Regarding the body area, the ankle and the knee were the anatomical areas most affected, with 40% and 16% respectively. Since the basketball game is dynamic that involves repeated sporting motion namely sudden changes in direction, side shifts, constant jumps, and the respective landings, these results were expected because of the higher physical demands of the lower limbs during basketball practice [15, 20]. Besides that, basketball players are usually tall individuals who present a higher laxity of ligaments and consequently less joint stability [20, 21].

Several studies reported similar data, showing that lower limbs were the most body area injured, especially the ankle and knee [2, 3, 9, 15–18, 20, 22, 23]. Owoeye et al. [15] reported that the knee had a injuries percentage of 41% and the ankle 22%, Foss et al. [16] revealed that the most common body part injured was the knee (67.9%) followed by the ankle (21.4%) and Borowski et al. [18] referred that the ankle/foot contributed by 39.7% of injuries and knee by 14.7%. Tummala et al. [22] reported 1,298 ankle injuries in men athletes during 868,625 athlete exposures, during the 10-year period, resulting in an injury rate of 1.49 injuries/1,000 athlete exposures and a total of 783,630 athlete exposures, totaling 950 injuries, in female players in the same period, resulting in an injury rate of 1.21 injuries/1,000 athlete exposures.

The high prevalence of ankle and knee injuries in basketball may be due to the multidirectional nature of basketball that includes constant acceleration and deceleration, causing athletes to have to perform rapid changes in direction, intermittent sprints, repetitive jumping, contacts with other players, and landing movements. Basketball is a vertical sport that requires 35–46 jumping and landing movements per game and changes of direction 2.0–2.82 s. [8, 22] Matthew and Delextrat [24] study reported that the players performed on average 652±128 movements per game, which corresponded to a change in activity every 2.82 s.

Repetitive jumping in basketball practice imposes recurring vertical ground reaction forces of up to 4 times body weight on the weight-bearing knee joint. Besides that, considering our sample (mostly adolescents), the ankle and knee injuries can be explained by the maturing neuromuscular system process that may be unable to maintain joint stability and around-joint control, leading to forces above the physiological threshold that can lead injuries on these joint structures [15].

Ankle sprain contributed by the most of injuries in our study and may occur when one player steps on the foot of another player, rolling the ankle inward, or when the player lands awkwardly, twisting the ankle, as well as when cutting, turning, or pushing off awkwardly [5]. Muscle injury (strain, contusion) and fracture contributed by 16% and 11% of all injuries in this study, respectively.

Our data about the type of injury are agreeing with several studies [3, 9, 15, 17, 18, 20, 23, 25]. Randazzo et al. [23] analyzed 4 128 852 injuries in pediatric basketball players that were treated in the emergency service and reported that the strains and sprains (44.8%) and fractures and dislocations (22.0%) were the most common injury observed.

Strategies for preventing ankle injuries can be included shoe design adaptations, use of foot orthotic devices (external ankle supports, including high-top shoes and ankle braces), and the appropriate protective equipment, taping, muscle strengthening, and neuromuscular training [8, 22, 26, 27]. Further research is needed to verify the effectiveness and correct use of ankle braces/taping and knee braces.

A training program that improves neuromuscular performance in proprioceptive control and postural sway integrated into athletic training period can be effective in reducing ankle and knee injuries [28, 29]. Taylor et al. [8] performed a systematic review and meta-analysis and found that the programs, with the objective of preventing injuries, reduced the incidence of lower extremity injuries (Odds ratio: 0.69; p≤0.001).

Regarding the mechanism of injury, the impact with another athlete was the most mechanism reported in our study (19%) following by the fall (14%). Similar results were obtained in other studies [9, 17]. Although rules restrict body contact, athletes resort to physical contact to gain and maintain positions close to the basketball hoop and the contact with another player is likely related to the mechanism of landing on an opponent after securing a rebound, so collisions with walls and floor, table supports and other athletes are sometimes unavoidable. The results of Owoeye et al. [15] and Moreira et al. [2] studies revealed that jumping or landing was the main mechanism of injury.

Basketball players may benefit from injury-prevention programs that safely simulate player contact. Besides that, better enforcement of rules and coaching that emphasizes less intentional fouls may mitigate the incidence of falls and another unintentional contact with the floor.

Most of the injuries in our study occurred during the training session, data that differ from those obtained in the study of Borowski et al. [18] that revealed that the injury rate was greater during the competition (3.27) than during practice (1.40). However, Moreira et al. [2] reported that, in most cases, sports injuries occurred during training (61.1%) and Foss et al. [16] referred that 53.6% of injuries occurred during practice and 46.4% occurred in a competition. These findings can be explained by the length of time in training that exposes the athlete to risk situations for longer duration time exposure.

This study also verified that the basketball players who trained more times a week had more probability of injury. The increased exposure may be related to an increased risk of injury due to repetitive and cumulative trauma. Future studies should confirm this result and establish new training strategies that include adequate rest between sessions.

Most athletes had to interrupt their training/competitions for more than 1 month due to injuries. In the Clifton et al. [17] study the injuries resulted in time loss of less than 1 week; the same was observed in Borowski et al. [18] study that showed that boys and girls returned to activity after injury most frequently in <1 week (55% and 483%, respectively). In Tummala et al. [22] study 44% of all men’s ankle injuries and 41% of women’s injuries resulted in a time loss of 3 days.

This study presents some limitations regarding the measuring instrument, as it is based on self-report, and the 12-month retrospective period may lead to memory bias, however, epidemiological studies to involve a representative sample of the population resort to these types of instruments. Future studies with the diagnostic evaluation of the injury by health professionals are suggested.

These results provide insight into the profile of Portuguese basketball injuries and can be used to develop preventative strategies. There is strong evidence that supports the effect of a neuromuscular balance training program on the improvement of the joint position sense and the postural sway.

## Conclusions

Data of this study showed a high prevalence of injuries in this analyzed stratified sample, being the ankle and knee the most injured body areas, the sprain the more prevalent type of injury, and the impact with another player the main mechanism of injury. Most of the injuries occurred in the training period and the player who trained more times per week have more probabilities of injury.
